# Detection of the GH analogue somatrogon in doping control urine samples by means of LC-HRMS/MS

**DOI:** 10.1038/s41598-025-96361-4

**Published:** 2025-04-16

**Authors:** Katja Walpurgis, Andreas Thomas, Alexandra Rauer, Bettina Majer, Mitsuhiko Sato, Masato Okano, Maneera Al-Jaber, Wadha Abushareeda, Cristian-Gabriel Arsene, Hans Geyer, Mario Thevis

**Affiliations:** 1 Institute of Biochemistry/Center for Preventive Doping Research, German Sport University Cologne, Am Sportpark Müngersdorf 6, 50933 Cologne, Germany; 2 Anti-Doping Laboratory, LSI Medience Corporation, Tokyo, Japan; 3 Anti-Doping Lab Qatar, Doha, Qatar; 4 Physikalisch-Technische Bundesanstalt (PTB), Braunschweig, Germany; 5 European Monitoring Center for Emerging Doping Agents (EuMoCEDA), Cologne/Bonn, Germany

**Keywords:** Affinity purification, Doping, Growth hormone analogue, LC-HRMS/MS, Tryptic digestion, Ultrafiltration, Urine, Peptide hormones, Metabolic pathways, Pharmacokinetics, Metabolomics

## Abstract

Somatrogon is a synthetic growth hormone (GH) analog, whose misuse in sports is prohibited at all times. As analytical approaches complementary to existing immunological GH detection methods were considered beneficial for its detection in doping control serum samples, a sensitive and specific qualitative mass spectrometric assay was recently developed, comprehensively characterized, and successfully employed to analyze authentic *in vivo* study samples. Within this follow-up project, this approach based on affinity purification, tryptic digestion, and LC-HRMS/MS was modified by implementing an additional ultrafiltration step to allow for the extraction of the intact drug and/or its metabolites from urine. Method validation demonstrated its specificity and sensitivity (LOD: 1 ng/mL), and the subsequent analysis of post-administration urine samples showed for the first time that intact (or at least GH receptor binding) somatrogon is excreted into urine and can be detected in most subjects for at least 96 h following injection. Consequently, anti-doping laboratories can also use urine to confirm the presence of somatrogon in athletes, where the GH differential isoform assay yielded atypical results in the corresponding serum specimens. Moreover, the latter assay also proved to be valuable as screening tool for the identification of urine samples potentially containing the GH analog.

## Introduction

**Fig. 1:**
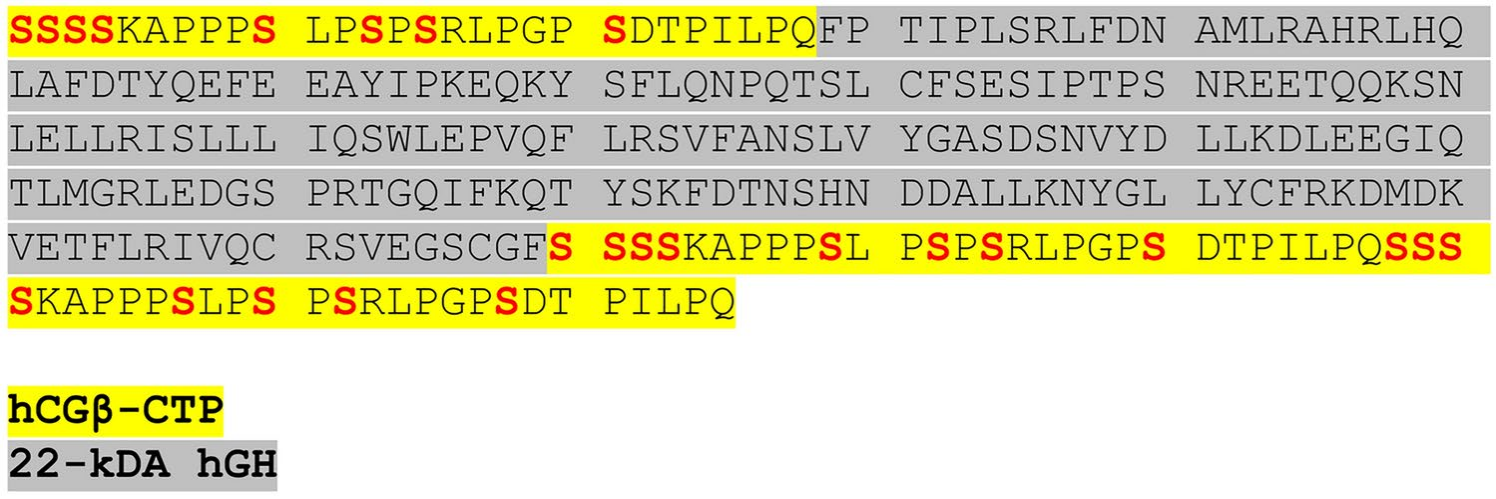
Amino acid sequence of somatrogon. One copy of the ß-hCG CTP (yellow) is attached to the N-terminus and two copies are fused to the C-terminus of 22 kDa hGH (grey). According to the literature, each CTP comprises 4–6 O-linked glycosylations at the serine residues highlighted in red. Moreover, up to 5 proline residues in each CTP can be hydroxylated.

According to the Prohibited List annually published by the World Anti-Doping Agency (WADA), the use of human growth hormone (hGH) in sports is prohibited both in- and out-of-competition^1^. Somatrogon (also referred to as MOD-4023 and Ngenla®) represents a novel recombinant long-acting GH analogue comprising the amino acid sequence of 22 kDa hGH where three copies of the C-terminal peptide (CTP) of the human chorionic gonadotropin β-subunit (β-hCG) are attached to the N- and C-Terminus (Fig. 1)^2–4^. The presence of these highly glycosylated CTPs results in a significantly extended biological half-life compared to recombinant hGH (rhGH), which allows for weekly instead of daily dosing. Due to the presumed lipolytic and anabolic effects similar to hGH, somatrogon was added to the WADA Prohibited List in 2022^5^. Its detectability with approved doping control immunoassays specific for hGH and hCG was recently evaluated by testing the assay’s cross reactivity with somatrogon reference material and analyzing somatrogon pre- and post-administration serum and urine samples^6^. Regarding hGH analysis, only Kit 2 of the hGH isoform differential immunoassays routinely employed by anti-doping laboratories was found to recognize the intact drug and therefore yielded elevated ratios between rhGH (Rec) and pituitary hGH (phGH, Pit) in the post-administration samples. Consequently, somatrogon misuse in sports can unfortunately remain undetected if Kit 1 is used as initial testing procedure (ITP) and if Kit 2 is employed, a confirmation with Kit 1 is not possible^7^. And, to date, it remains to be clarified if the hGH biomarkers approach offers a detection option. For that reason, a specific mass spectrometry (MS)-based somatrogon assay suitable for confirmation purposes was recently developed^6^ and allowed the detection of the drug in serum for at least 96 h following administration. While neither the hCG ITP specific for total hCG, nor the confirmation procedure (CP) for intact hCG showed any cross reactivity with somatrogon, several post-administration urine specimens returned positive when analyzed with the ITP, indicating that immunoreactive metabolites/fragments of the fusion protein might be present in urine. According to the literature^8,9^, intact somatrogon with an approximate molecular mass of 40 kDa is not expected to be excreted into urine but rather metabolized by proteolysis. However, neither the drug’s excretion, nor its metabolism have been investigated so far. As the use of urine instead of serum as biological matrix would be advantageous for sports drug testing regarding sample collection and transportation as well as analytical aspects such as the available sample volume and achievable analytical spectrum^10^, the aim of this follow-up research project was to investigate the urinary excretion of somatrogon and develop a mass spectrometric assay for its detection from urine. For that purpose, the existing method for the extraction and analysis of serum specimens^6^ was modified by including an additional ultrafiltration step, optimized, comprehensively characterized, and applied to authentic somatrogon *in vivo* study urine samples as proof-of-concept. Moreover, the applicability of existing analytical options such as the hGH isoform differential immunoassays was evaluated.

## Material & methods

### Reference material, chemicals & consumables

Somatrogon reference material was from Pfizer (NYC, NY) and U-^15^N-labelled 22 kDa GH from Novo Nordisk (Bagsværd, Denmark) was used as internal standard (ISTD) to monitor sample extraction and analysis.

Amicon® Ultra-4 centrifugal filter units (cut-off: 10 kDa) were bought from Merck (Darmstadt, Germany) and magnetic beads for affinity purification were prepared by using NHS Mag Sepharose^TM^ from Cytiva (Marlborough, MA) and a recombinant human GH receptor (GHR) Fc Chimera from R&D Systems (Minneapolis, MN). Reagents required for protein reduction and alkylation were dithiothreitol (DTT) and iodoacetamide (IAA), and purchased from Merck and Thermo Fisher Scientific (Waltham, MA). Sequencing grade modified trypsin (V5111) was obtained from Promega (Madison, WI), and all other chemicals/solvents were bought from Merck (Darmstadt, Germany) and of analytical grade.

NuPAGE^TM^ 12% Bis-Tris mini gels (10-well, 8 × 8 × 0.1 cm), MOPS (3-(N-morpholino) propanesulfonic acid) running buffer, LDS (lithium dodecyl sulfate) sample buffer, and antioxidant were also from Thermo Fisher Scientific. PVDF (polyvinylidene difluoride) membranes and a goat anti-human IgG secondary antibody conjugated to HRP were obtained from Merck and the SuperSignal™ West Femto Maximum Sensitivity Substrate employed for Western blot detection was also bought from Thermo Fisher Scientific.

Immunoassays for the measurement of hGH isoforms (hGH LIA Kit 1 and 2) were purchased from CMZ-Assays (Berlin, Germany).

### Samples

#### Somatrogon administration study

As described elsewhere^6^, three healthy males (B01–B03, age: 20–23, body weight: 53–58 kg) and three healthy females (B04–B06, age: 21–33, body weight: 57–68) received a single subcutaneous injection of somatrogon at a dosage of 0.66 mg/kg. Both serum and urine specimens were collected before and up to 96 h following administration, and stored at –20 °C until analysis. For serum collection, BD Vacutainer® serum separator tubes II (SST II, BD, Becton Dickinson GmbH, Heidelberg, Germany) were used according to the manufacturer’s instructions. Before study enrolment, ethical approval was obtained from the Institutional Review Board (IRB) of the LSI Medience Corporation (MS/Shimura 22–10), whose guidelines and regulations were applied to the entire research project and all included methods. All participants provided written informed consent and the study was performed in accordance with the Declaration of Helsinki.

#### Other samples

For method development and characterization, urine samples were collected from 10 healthy volunteers (5 males and 5 females). All participants provided written informed consent, and the study was approved by the local ethical committee (DSHS No. 139/2021), whose guidelines and regulations were applied to this research project. Sample collection, processing and analysis were conducted in accordance with the Declaration of Helsinki. For the determination of certain validation parameters such as linearity, stability, carryover & recovery, a mix-urine was prepared by mixing equal proportions of these samples.

Moreover, a total of 95 doping control routine samples (females: n = 32, males: n = 63) were analyzed to assess levels of common urinary hGH concentrations. Of these samples, 63% (≡ 60) were collected in-competition.

### Detection of somatrogon in urine by means of ultrafiltration, affinity purification, tryptic digestion and LC-HRMS/MS

#### Magnetic beads preparation

As in the previous study^6^, GHR-Fc-conjugated magnetic beads were employed for affinity purification. For method development, validation, and the analysis of the administration study samples, ten batches were prepared in parallel according to the manufacturer’s recommendations as follows: For each batch, 25 µL of NHS Mag Sepharose^TM^ were transferred to an Eppendorf tube and the storage solution was removed by using a magnetic rack. Following equilibration with 500 µL of ice cold 1 mM HCl, magnetic beads were incubated with 5 µg of GHR-Fc diluted in 50 µL of coupling buffer (0.2 M NaHCO_3_, 0.5 M NaCl, pH 8.3) for 30 min at 1200 rpm and room temperature (RT). The supernatant was removed and magnetic beads were consecutively washed with 500 µL of blocking buffer A (50 mM Tris–HCl, 1 M NaCl, pH 8.0) and blocking buffer B (50 mM glycine, 1 M NaCl, pH 3.0) for a total of three times. Thereby, the second washing step with blocking buffer A was carried out for 15 min at RT on a rotating sample mixer. Subsequently, magnetic beads were equilibrated twice in 500 µL of phosphate-buffered saline (PBS; 1 tablet per 200 mL of deionized water: 0.01 M phosphate buffer, 0.0027 M potassium chloride and 0.137 M sodium chloride, pH 7.4), reconstituted with 300 µL of PBS, pooled and stored at 4 °C until usage.

#### Sample extraction

**Fig. 2:**
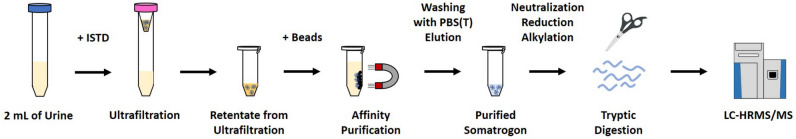
Method overview.

As shown in Fig. 2, 2 mL of each urine sample were aliquoted into a 15 mL Falcon tube and fortified with 50 ng of ISTD (10 µL of a solution containing 5 µg/mL of U-^15^N-labelled 22 kDa GH). To precipitate urinary sediments, samples were centrifuged for 5 min at 4,000 × *g* and supernatants transferred to Amicon® Ultra-4 centrifugal filter units with a cut-off of 10 kDa. Following centrifugation for 10 min at 4,000 × g, retentates were washed with 4 mL of PBS (15 min, 4,000 × *g*) and mixed with 300 µL of GHR-Fc-conjugated magnetic beads. Affinity purification was then carried out for approximately 60 min at RT on a rotating sample mixer. To remove urinary proteins non-specifically bound to the surface of the magnetic beads, washing steps with once 500 µL of PBST (0.02% Tween 20) and twice 500 µL of PBS were conducted. Then, ligands specifically bound by the GHR were eluted with 50 µL of 3% acetic acid (HAc) for 15 min at 1200 rpm and RT.

To allow for their re-usage, magnetic beads were washed with 300 µL of 3% HAc for a total of three times, once with 500 µL of PBST, and twice with 500 µL of PBS. Then, they were resuspended in 300 µL of PBS and returned to the magnetic beads stock solution stored at 4 °C.

#### Tryptic digestion

Sample extracts obtained from affinity purification were neutralized with 25 µL of 2 M NH_4_HCO_3_ and subjected to protein reduction with 750 nmol of DTT (7.5 µL of a 0.1 M solution in 100 mM NH_4_HCO_3_) for 30 min at 60 °C and 900 rpm and alkylation with 1,875 nmol of IAA (7.5 µL of a 0.25 M solution in 100 mM NH_4_HCO_3_) for 30 min at RT in the dark. Subsequently, 800 ng of trypsin (20 µL of a solution with 40 µg/mL in 50 mM NH_4_HCO_3_) and 10 µL of acetonitrile (ACN) were added and samples incubated overnight at 37°C and 500 rpm. To stop tryptic digestion, 5 µL of glacial acetic acid were added. Then, samples were transferred to polypropylene HPLC vials and subjected to LC-HRMS/MS analysis.

#### LC-HRMS/MS

For LC-HRMS/MS measurements, an Orbitrap Exploris™ 480 (Thermo Fisher Scientific) interfaced with a Vanquish UHPLC was employed. The LC system was equipped with an Accucore™ Phenyl-Hexyl trapping column (3 × 10 mm, 2.6 μm; Thermo Fisher Scientific) and a Poroshell EC-C18 column analytical column (3 × 50 mm, 2.7 μm; Agilent Technologies, Santa Clara, CA, USA), and the column temperature was set to 30 °C. Of each sample extract, 10 µL were injected and the autosampler temperature was 10 °C. The following LC gradient using 0.1 formic acid in water with 1% dimethyl sulfoxide (DMSO) as solvent A and 0.1 formic acid in ACN with 1% DMSO as solvent B was run with a flow rate of 0.4 mL/min: 0–2 min isocratic trapping with 5% B1, 2–8 min 5–40% B, 8–10 min 40–80% B, 10–15 min re-equilibration with 5% B. The solvents employed for trapping were 0.1% formic acid in water (A1) and 0.1% formic acid in ACN (B1).

The MS system was operated in positive ionization mode with an ionization voltage of 4 kV, and the temperature of the ion transfer tube was set to 320 °C. A Full Scan from *m/z* 400–2400 was recorded at a resolution of 30,000 full width at half maximum (FWHM) at *m/z* 200, and targeted selected ion monitoring (tSIM) experiments with an isolation window of *m/z* = 3 and a resolution of 60,000 FWHM at *m/z* 200 were conducted using an inclusion list with the accurate mass-to-charge ratios for the most abundant charge states of the target peptides (Table 1). Moreover, data-dependent MS/MS (ddMS^2^) scans with a mass isolation window of *m/z* = 2, a first mass of *m/z* = 200, and an intensity threshold of 1.0E4 were recorded at a resolution of 15,000 FWHM at *m/z* 200 by using a higher-energy collisional dissociation (HCD) collision energy (CE) of 30% and nitrogen obtained from a N_2_-generator (CMC, Eschborn, Germany) as collision gas. For the evaluation of the acquired MS data, Thermo Xcalibur Software (Version 4.0.27.10, 2015) was used.

**Table 1:** Target peptides for LC-HRMS/MS.

Analyte	Tryptic peptide #	Amino acid sequence	Amino acid positions	Modification(s)	m/z*	Charge State
Somatrogon	T_1_–T_3_	SSSSKAPPPSLPSPSRLPGPSDTPILPQFPTIPLSR	1–36	4 x NANA-Gal-GalNAc (NT-4G)	1584.99	4
				4 x NANA-Gal-GalNAc + HP (NT-4G-HP)	1588.99	4
				5 x NANA-Gal-GalNAc (NT-5G)	1749.04	4
				5 x NANA-Gal-GalNAc + HP (NT-5G-HP)	1753.04	4
	T_3_	LPGPSDTPILPQFPTIPLSR	17–36	-	1074.10	2
	T_23_	SVEGSCGFSSSSK	212–224	-	659.78	2
22 kDa-hGH	T_2_	LFDNAMLR	9–16	-	490.26	2
	T_4_	LHQLAFDTYQEFEEAYIPK	20–38	-	1171.57	2
	T_10_	SVFANSLVYGASDSNVYDLLK	95–115	-	1131.57	2
U-15N-22kDa-hGH (ISTD)	T_1_	FPTIPLSR	1–8	U-^15^N-Labelling	471.26	2
	T_4_	LHQLAFDTYQEFEEAYIPK	20–38	U-^15^N-Labelling	789.36	3
	T_9_	ISLLLIQSWLEPVQFLR	78–94	U-^15^N-Labelling	1039.57	2

*Most abundant isotopes, cysteine residues alkylated with iodoacetamide. *NT* N-terminus, *4G* 4 Glycosylations, *5G* 5 Glycosylations.

### Method validation

Method validation was conducted according to current WADA guidelines^11^ as follows:

- **Selectivity:** To demonstrate the selectivity of the method, 10 different blank urine samples were analyzed as described above.
- **Reliability:** The reliability of the approach was investigated by preparing 10 different urine specimens fortified with 10 ng/mL of somatrogon according to the presented protocol. 
- **Limit of detection (LOD):** Somatrogon was spiked into 3 x 6 different urine samples at concentrations of 0.1, 0.5 and 1 ng/mL, and they were extracted and analyzed according to the protocol above. Following LC-HRMS/MS, a detection rate of >95% was applied to estimate the LOD.
- **Linearity:** Linearity was assessed by analyzing 10 mix-urine specimens fortified with 0, 1, 2.5, 5, 7.5, 10, 25, 50, 75 and 100 ng/mL of somatrogon. Absolute peak areas were used to construct a calibration curve and linearity was determined by regression analysis.
- **Robustness:** The robustness was investigated by analyzing 10 different urine samples containing 10 ng/mL of somatrogon with a slightly modified sample preparation protocol: Instead of overnight digestion, proteolysis was only conducted for 1 h at 37 °C.
- **Carryover (Magnetic Beads):** Sample carryover of the GHR-Fc conjugated magnetic beads was investigated as follows: Two mix-urine samples were fortified with 100 and 500 ng/mL of somatrogon and prepared as described above. Following magnetic beads elution, they were washed with 3 x 300 µL of 3% HAc and of each wash fraction, 50 µL were subjected to neutralization, protein reduction and alkylation, tryptic digestion, and LC-HRMS/MS as described above.
- **Carryover (LC-HRMS/MS):** The risk for sample carryover during LC-HRMS/MS measurements was assessed by injecting a blank urine extract immediately after the extract of a sample fortified with 100 ng/mL of somatrogon.
- **Stability: **Linearity sample extracts were stored in the autosampler of the LC-MS system at a temperature of 10 °C for a total of 3 days and subsequently re-analyzed to determine analyte stability.
- **Recovery:** To determine the recovery of ultrafiltration and affinity purification, the following mix-urine samples were prepared: Two sets (n = 3) of blank urine samples, one set (n = 3) of urine samples containing 100 ng/mL (≡ 200 ng) of somatrogon. All samples were fortified with the ISTD and subjected to ultrafiltration and affinity purification as described above. To one set of blank urine samples, 100 ng/mL (≡ 200 ng) of somatrogon were added following ultrafiltration, to the other set after affinity purification. Finally, the recovery of the ultrafiltration alone and in combination with affinity purification was determined by comparing the normalized peak areas of the samples fortified with the target analyte before and following concentration/extraction.

#### Stability of somatrogon in urine

To evaluate the stability of somatrogon in urine, two fresh urine specimens (1 male, 1 female) were spiked with the target analyte at a concentration of 25 ng/mL, and aliquots of 2 mL were stored for 1, 3, 7, and 14 days (d) both at 4°C and RT. Blank and reference samples of each volunteer were directly stored frozen at –20 °C. After the respective storage period at 4 °C/RT, stability samples were frozen at –20 °C until extraction and analysis.

#### Analysis of somatrogon *in vivo* study samples

As proof-of-concept, somatrogon pre- and post-administration urine samples were analyzed as described above. To roughly estimate the analyte concentrations in the samples, a single point calibrator fortified with 25 ng/mL of somatrogon was prepared with each sample batch.

### Characterization of urinary somatrogon

To characterize the urinary somatrogon (derivative(s)) detected with the LC-HRMS/MS assay, the following Western blotting experiment was conducted: Both a pre- and post-administration urine sample were subjected to ultrafiltration and affinity purification as described above, and 20 µL of the resulting sample extracts were mixed with 7 μL of LDS sample buffer and 3 μL of DTT. Similarly, 40 ng each of somatrogon and the ISTD were prepared as references. To achieve protein denaturation and reduction of disulfide bonds, all samples were heated for 10 min at 70°C. Proteins were separated on a 12% Bis–Tris mini gel by means of SDS-PAGE (~ 90 min, 125 V) with MOPS running buffer and antioxidant. Following electrophoresis, separated proteins were transferred to a PVDF membrane by using a semi-dry transfer unit (GE Healthcare; 1 mA/cm^2^, 45 min) and transfer buffer adapted from Bjerrum and Schäfer-Nielsen^12^ (39 mM glycine, 48 mM tris, 0.0375% SDS, 20% ethanol). Membrane blocking was conducted with 5% (w/v) skim milk in PBST-2 (0.1 M NaCl, 0.08 M Na_2_HPO_4_, 0.02 M NaH_2_PO_4_ × 1 H_2_O, and 0.1% Tween 20) overnight at 4 °C. Instead of using a primary antibody, the membrane was incubated with 1 µg/mL of GHR-Fc in 1% skim milk (in PBST-2) for 1 h at RT and subsequently washed with PBST-2 for a total of three times (1 × 20 min, 2 × 10 min). As secondary antibody, a goat anti-human IgG HRP conjugate (Sigma-Aldrich; 1:10 000 in 1% skim milk) was used (1 h, RT). After repeated washing in PBST-2 (1 × 20 min, 2 × 10 min), luminol was added as substrate (5 min, RT) and chemiluminescence images were recorded with an ImageQuant LAS 4000 CCD camera system (GE Healthcare).

### Analysis of urine samples with the hGH isoform differential immunoassays

Even though the hGH isoform differential immunoassays routinely employed by anti-doping laboratories were developed for serum, their principal applicability to somatrogon testing in urine was evaluated within this research project. For that purpose, selected pre- and post-administration urine specimens were analyzed in duplicate with GH isoform Kits 1 and 2 according to the manufacturer’s instructions and luminescence measurements were conducted on a Centro LB 963 luminometer from Berthold Technologies (Bad Wildbad, Germany). Samples with concentrations above 12.5 ng/mL were diluted with blank urine and re-analyzed.

To determine urinary hGH reference levels, a total of 95 doping control routine urine samples collected in- and out-of-competition were additionally analyzed with both kits.

## Results and discussion

### Mass spectrometric detection of somatrogon in urine

**Fig. 3:**
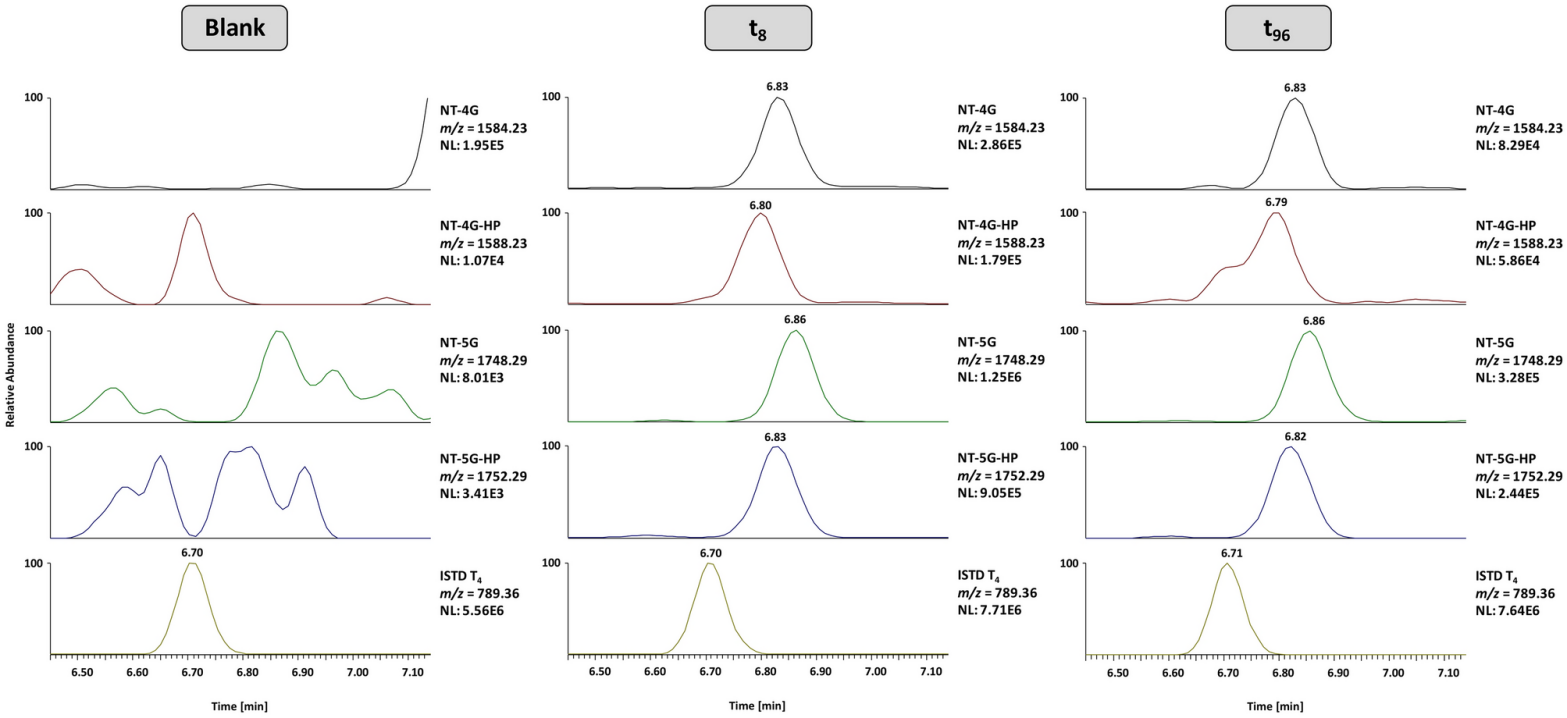
Extracted ion chromatograms of the main target peptides NT-4G, NT-4G-HP, NT-5G, and NT-5G-HP and the ISTD of a urine specimen collected prior to somatrogon injection, as well as two post-administration urine samples collected after 8 and 96 h (volunteer B02).

The protein composition of urine is determined by the size-, charge-, and shape-selectivity of the glomerular barrier between blood and primary urine, as well as reabsorption processes in the renal tubules^13,14^. Here, the 150–180 L of primary filtrate formed in the glomerulus everyday are highly concentrated so that, ultimately, less than 1% is excreted as urine. Additionally, urine often contains proteins originating from the urinary tract or post-renal contaminations, and different (pathological) conditions such as *Diabetes* and strenuous exercise were found to have significant effects on renal protein excretion^15^. Additionally, urinary proteases can be present and degrade large proteins especially at low pH^16,17^. Therefore, the use of urine as biological matrix for protein testing in clinical settings or doping control analysis can be associated with different challenges such as the highly variable protein composition and low urinary concentrations of proteins with a high molecular mass. Consequently, the first step required for targeted protein analysis is sample concentration. Within this research project, two different strategies were tested: solid-phase extraction (SPE) using Waters Oasis HLB cartridges with 3 ccm and 60 mg of sorbent followed by evaporation in a concentrator under reduced pressure^18^, (protocol see Supplementary Methods 1), and ultrafiltration with Amicon® Ultra-4 centrifugal filter units with a cut-off of 10 kDa. Both approaches were subsequently combined with affinity purification, tryptic digestion, and LC-HRMS/MS, and found to allow for the detection of the four somatrogon target peptides from urine fortified with the drug (Supplementary Fig. 1). As the ultrafiltration protocol was not only significantly shorter but also provided a better sensitivity, it was eventually chosen for protein concentration and further optimized. After successfully testing the method’s applicability using somatrogon-spiked urine specimens, it was applied to exemplary somatrogon pre- and post-administration urine samples, and the four main target peptides corresponding to different isoforms of the glycosylated and hydroxylated N-terminus T_1-3_ (Fig. 3) as well as the somatrogon-specific peptide T_23_ and three peptides corresponding to both somatrogon (T_4_, T_6_ & T_12_) and 22-kDa hGH (T_2_, T_4_ & T_10_) were detected for up to 96 h following injection. Additionally, several other tryptic peptides located in the 22-kDa hGH proportion of the fusion protein (T_6_, T_8,_ T_11,_ T_15_ & T_16_) were identified (Supplementary Table 1). Both the successful affinity purification with the GHR and the high sequence coverage of somatrogon suggest that the intact (or nearly intact) fusion protein is present in urine. This is contrary to the basic information on the drug’s metabolism/elimination shared by the manufacturer^9^ and the European Medicines Agency^8^, stating that the drug is (probably) degraded by proteases and not excreted into urine.

### Method characterization

**Fig. 4:**
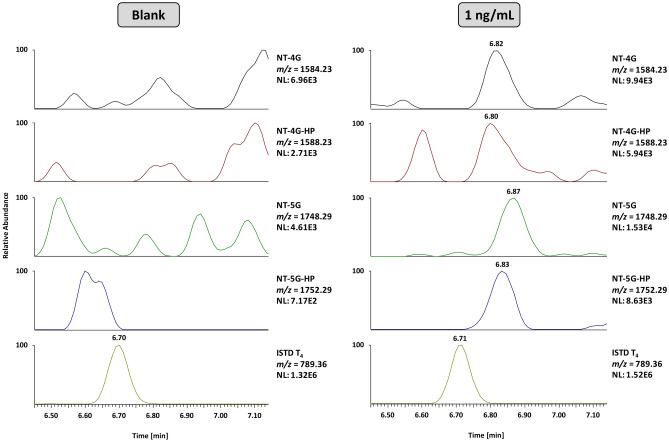
Exemplary extracted ion chromatograms of the main target peptides NT-4G, NT-4G-HP, NT-5G, and NT-5G-HP and the ISTD of a blank and a urine sample fortified with 1 ng/mL of somatrogon.

The applicability of the method to sports drug testing was demonstrated by a comprehensive validation conducted according to current WADA guidelines^11^. As shown in Table 2, the approach was found to be highly specific, reliable (at 10 ng/mL), and linear from 1 to 100 ng/mL. At higher analyte concentrations, a saturation of affinity purification was observed (data not shown). The detection limit was estimated as 1 ng/mL, however, sensitivity can be increased by using a higher sample and/or injection volume. Figure 4 shows exemplary extracted ion chromatograms of a blank and a urine sample containing 1 ng/mL of somatrogon. No relevant interferences were observed in the blank specimen and the main target peptides NT-4G, NT-4G-HP, NT-5G and NT-5G-HP were unambiguously detected in the fortified sample. To demonstrate the robustness of the method, the incubation time for tryptic digestion was significantly shortened from overnight (~ 18 h) to 1 h. In 10 different urine samples spiked with 10 ng/mL of somatrogon, all target peptides were unambiguously detected, however, the intensities of tryptic peptides originating from more centrally located amino acid sequences of somatogon (T_23_) and the ISTD (T_4_, T_9_) were found to be significantly lower than after a longer proteolysis. This can probably be explained by an incomplete cleavage of the target protein. Both for affinity purification and LC-HRMS/MS, carryover experiments were conducted, demonstrating that magnetic beads can be re-used when thoroughly washed with acetic acid and PBS buffer. Carryover during LC-HRMS/MS was below 1%, and urine sample extracts were found to be stable in the autosampler for at least 3 days. The recoveries of ultrafiltration alone and in combination with affinity purification were approximately 78% and 27%, indicating that most analyte losses occur during sample extraction with GHR-Fc conjugated magnetic beads.

**Table 2:** Results of method validation

Validation parameter:	n	Concentration(s) [ng/mL]:	Somatrogon
Selectivity	10	–	0/10
Reliability of Detection	10	10	10/10
LOD	6	0.1	0/6
	6	0.5	1/6
	6	1	6/6
Linearity	10	1–100	R² ≥ 0.989
Robustness (1 h digest)	10	10	10/10
Carryover (Magnetic Beads)	2	100/500	Yes
Carryover (LC-HRMS/MS)	2	100/500	< 1%
Sample Extract Stability	8	1–100	≥ 3 days
Recovery (Magnetic Beads)	9	100	~ 27%

### Stability of somatrogon in urine

While doping control blood samples have to be transported to the laboratory refrigerated and temperature controlled within a certain period of time^19^, no specific rules apply to urine samples. To ensure compatibility of somatrogon urine testing with doping control routine procedures, the analyte’s stability at 4 °C and RT in relation to reference samples stored at –20 °C was investigated over a period of 14 days. At both temperatures, the drug was found to be stable for at least 2 weeks (Supplementary Fig. 2). Consequently, a transportation at ambient temperature and refrigerated storage for a few days each should have no negative effects on the detectability of the protein drug. A potential degradation of the drug during storage at –20 °C was not investigated.

### Analysis of administration study samples

In sports drug testing, the analysis of authentic *in vivo* study specimens is of utmost importance to provide proof-of-concept for the detectability of a prohibited substance or its metabolites/markers in a real sample by using a certain analytical approach. As somatrogon was originally not expected to be excreted into urine, the analysis of the available urine samples can additionally provide valuable insights into the metabolism and elimination behavior of the drug. In four subjects, urine samples collected between 2 and 96 h following injection were found to contain detectable amounts of at least some somatrogon target peptides (Table 3). The extracted ion chromatograms of a urine sample collected prior to somatrogon injection, as well as two post-administration specimens obtained after 8 and 96 h from subject B02 are shown in Fig. 3. While none of the target peptides was observed in the pre-administration sample, all somatrogon isoforms were clearly detectable for at least four days following injection. In one female volunteer (B04), the protein drug could not be detected in the urine samples obtained at time points t_2_, t_4_, t_6_, and t_8_, and in the female subject B05, only sample t_12_ yielded traces of peptides NT-5G and NT-5G-HP. In the corresponding serum samples of this volunteer^6^, somatrogon concentrations were found to be significantly lower than in the other subjects (< 500 ng/mL). So overall, the urinary elimination of somatrogon appeared to be highly variable, suggesting that there might be inter-individual differences regarding the drug’s bioavailability, protein metabolism and/or renal elimination. As the LC-HRMS/MS assay can only detect somatrogon-derived species bound by the GHR, urinary metabolites/fragments lacking larger proportions of the molecule are not compatible with this analytical approach. Additionally, the detectability of the ISTD was found to vary strongly between samples, which indicates that certain matrix characteristics such as the presence of urinary proteases or very high/low amounts of other proteins can interfere with the extraction and/or detection of the drug. Also, the effectiveness of tryptic digestion is likely to be affected by the overall protein concentration and the presence of inhibiting molecules.

**Table 3:**
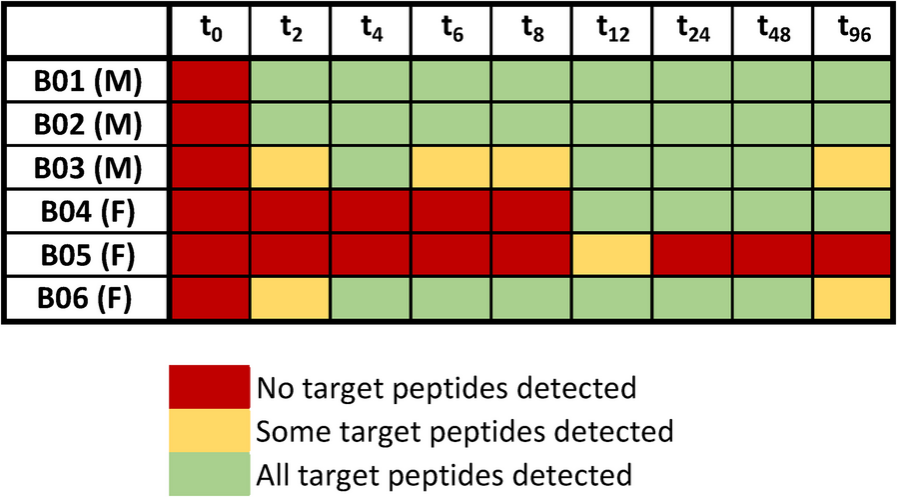
Detectability of somatrogon in pre- and post administration urine samples

### Characterization of urinary somatrogon

**Fig. 5:**
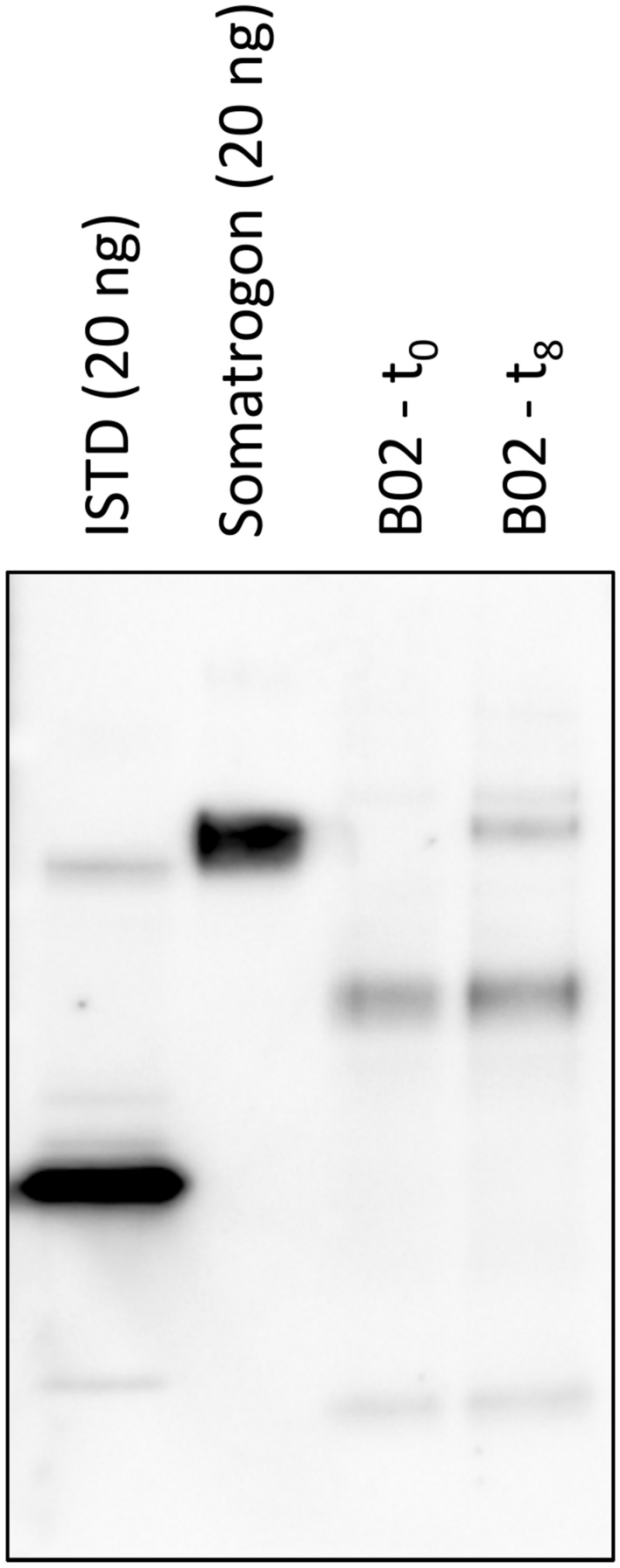
Western blot image of somatrogon pre- and post-administration urine sample extracts detected with the GHR-Fc. The unedited Western blot is shown in Supplementary Fig. 3.

To further characterize the urinary somatrogon (derivative(s)) detected by means of LC-HRMS/MS, pre- and post-administration sample extracts and reference material for the ISTD/22 kDa hGH and somatrogon were electrophoretically separated and subjected to Western blot analysis using GHR-Fc instead of a primary antibody and a goat anti-human IgG HRP conjugate for detection purposes. As shown in Fig. 5, a band with the same apparent molecular weight (MW) as the somatrogon reference material (~ 50 kDa) was detected in the extract of the urine specimen collected 8 h following somatrogon injection, indicating that—as already suggested by the LC-HRMS/MS data—intact or nearly intact somatrogon is excreted into urine. In both the pre- and post-administration sample extracts, several other bands were observed at apparent MWs of ~ 15, ~ 35, and ~ 55 kDa. An additional experiment in which a membrane with pre- and post-administration sample extracts and reference standards was incubated with the secondary antibody only (data not shown) demonstrated that these bands are artefacts caused by direct binding of the goat anti-human IgG antibody-HRP conjugate. Therefore, it can be assumed that these signals are derived from co-purified urinary IgG (fragments) and GHR-Fc co-eluted from the GHR-Fc magnetic beads. Overall, these results appear plausible as somatrogon metabolites or fragments lacking larger proportions of the hGH amino acid sequence would probably not be bound by the receptor. However, intact urinary somatrogon is very unlikely to be the cause for the positive hCG findings observed in some of the post-administration urine specimens as the hCG ITP specific for total hCG showed no cross-reactivity with the drug^6^. Therefore, it can be assumed that also smaller, immunoreactive somatrogon fragments/metabolites are renally eliminated, which do not interact with the GHR. Their identification and characterization will be the subject of a follow-up research project.

### Analysis of urine samples with the hGH isoform differential immunoassays

**Fig. 6:**
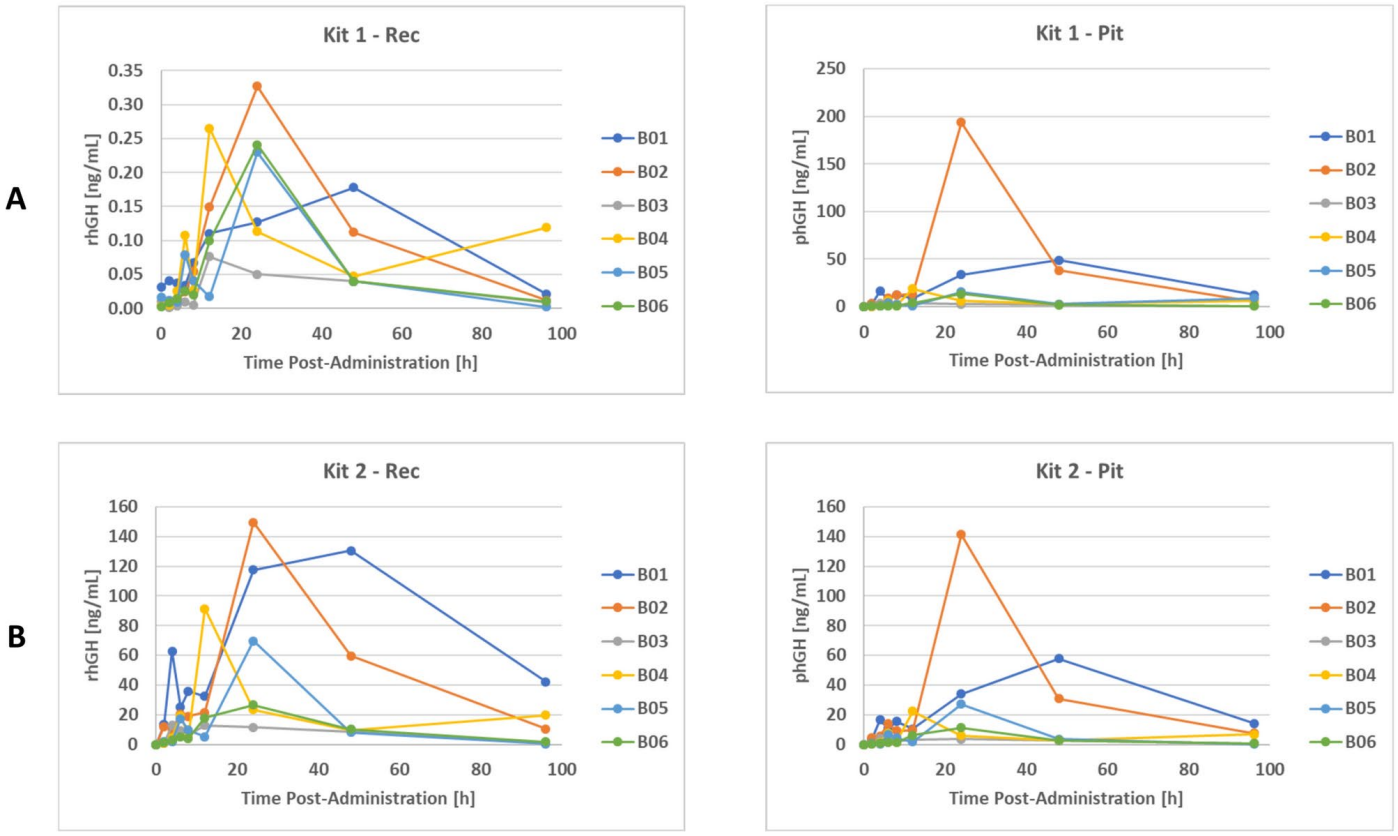
Detection of somatrogon in urine using the GH differential isoform assay Kit 2.

In routine sports drug testing, serum is the preferred biological matrix for hGH detection as only 0.01–0.001% of the amounts circulating in the blood are renally eliminated, resulting in urinary concentrations of only a few pg/mL or even less^20,21^ . For serum analysis, two different kits (“1” and “2”) each comprising two sandwich-type chemiluminescence immunoassays for the quantification of 22 kDa hGH/rhGH (“Rec”) and all pituitary hGH isoforms (“Pit”) are employed by anti-doping laboratories^7,23^. As the exogenous administration of rhGH results in a reduced secretion of pituitary hGH, the resulting Rec/Pit ratio can provide evidence for hGH doping. Urinary GH levels should usually be below the detection limit of the GH isoform differential immunoassay Kit 2, which has an analytical sensitivity of 22 pg/mL^22^ and proved to be compatible with somatrogon detection in serum^6^. As the administration of the GH analogue was found to result in urine levels of at least several ng/mL, the amounts of rhGH measured with Kit 2 in the post-administration urine specimens should be equivalent to the urinary concentrations of somatrogon. As shown in Fig. 6B, the drug could be detected in all post-administration urine samples and maximum levels between 13 and 150 ng/mL were observed 12–48 h following injection. Again, a high inter-individual variability regarding the urinary elimination of somatrogon was observed. For two female volunteers (B04 and B05), the results obtained with the immunoassay differed significantly from those determined by LC-HRMS/MS analysis, where somatrogon was undetectable in several/most samples. This can probably be explained by the presence of somatrogon metabolites/fragments still bound by the antibody used in Kit 2 but not by the GHR used for affinity purification prior to LC-HRMS/MS. The complementary analysis of the samples with the Pit assay of Kit 2 also revealed a post-dose response following somatrogon injection, however, the detected amounts were significantly lower than the concentrations estimated with the Rec assay. Most likely, this can be attributed to the significantly lower cross-reactivity (ca. 17.5% vs. ca. 113%) of the anti-phGH antibody employed in Kit 2 with intact somatrogon demonstrated in the previous study^6^.

Even though the Rec assay of GH isoform differential immunoassay Kit 1 cannot detect intact somatrogon^6^, the pre- and post-administration urine samples were also analyzed with this Kit as potential urinary metabolites could possibly be bound by the employed anti-rhGH capture antibody. And indeed, both the Rec and Pit assay yielded post-dose responses in the urine samples (Fig. 6A). While the observed increases in the urinary somatrogon concentrations determined with the Pit assay were in a similar range as measured with Kit 2, which is in accordance with the comparable cross-reactivities of the anti-phGH capture antibodies^6^, the amounts estimated with the Rec assay were significantly lower by a factor of 111–1695. A potential explanation could be the presence of a low-abundant metabolite in which the epitope located at the C-terminus of 22 kDa hGH^23^ is not shielded by the β-hCG CTP present in intact somatrogon.

Additionally, a total of 95 doping control routine urine specimens were analyzed with Kit 1 and 2 to determine urinary hGH reference levels. In about 70% of the samples, rhGH concentrations were below the kits’ detection limits of 21 and 22 pg/mL^22,24^ (Supplementary Table 2). In ca. 18% of the samples, the measured rhGH levels were found to be higher than 100 pg/mL and in 6% even above 1 ng/mL. With regard to existing literature on urinary hGH excretion^20,21^, these values appear to be unexpectedly high, however, it should be considered that the urinary excretion of proteins can be significantly affected by strenuous exercise^15^ and that the employed immunoassays were not developed for the analysis of urine specimens. While the estimated rhGH/somatrogon concentrations in the post-administration samples significantly differed between Kit 1 and 2 (Rec2/Rec1 = 111–1695), comparable concentrations resulting in Rec2/Rec1 ratios between 0.8 and 2.4 were determined for the reference samples containing detectable amounts of rhGH (≥ 0.021 ng/mL with Kit 1 and ≥ 0.022 ng/mL with Kit 2). Therefore, besides an unusually high urinary hGH concentration, also this value could be evaluated as screening tool for the identification of urine samples potentially containing somatrogon, which should be subjected to further analysis by means of LC-HRMS/MS.

## Conclusions

After the GH analog somatrogon was added to the WADA Prohibited List in 2022^1,5^, the Japanese anti-doping laboratory evaluated its detectability with approved doping control immunoassays specific for hGH and hCG^6^. Unfortunately, only Kit 2 of the hGH isoform differential immunoassays was found to detect the intact drug in serum and neither Kit 1, nor the hCG ITP specific for total urinary hCG and CP for intact urinary hCG showed any cross reactivity with the GH analog. Therefore, a qualitative method for the specific detection of somatrogon from doping control serum and plasma samples based on affinity purification with GHR-Fc conjugated magnetic beads, tryptic digestion, and LC-HRMS/MS was recently developed in a joint project. The approach was comprehensively characterized and the analysis of authentic post-administration serum samples demonstrated that the drug can be detected in serum for at least 96 h following a single-dose injection. This approach can be employed to confirm the presence of somatrogon in serum samples where only Kit 2 of the hGH isoform assay yielded an abnormally high Rec/Pit ratio. But despite a lacking cross reactivity with intact somatrogon, some of the post-administration urine samples were characterized by elevated concentrations of total hCG, and it can be assumed that urinary metabolites or degradation products of somatrogon comprising the CTP of β-hCG were recognized by the kit. Therefore, the protocol for the specific detection of somatrogon from serum/plasma was modified within this follow-up research project by implementing an initial ultrafiltration step for urine concentration, and tested on exemplary post-administration urine samples. Surprisingly, either the intact drug or a large metabolite/derivative still binding to the GHR appeared to be present in urine and could be detected in most volunteers at for least 96 h following administration. The method was comprehensively characterized and allowed a highly specific and sensitive detection of somatrogon from urine down to a concentration of 1 ng/mL.

Additionally, the applicability of the hGH isoform assay to urinary somatrogon detection was evaluated and urinary rhGH concentrations measured with Kit 2 increased from a few pg/mL up to 150 ng/mL following somatrogon injection. By contrast, the lacking cross-reactivity of the anti-rhGH antibody employed in Kit 1 with the intact GH analog resulted in only slightly elevated urine levels up to 265 pg/mL, which can potentially be explained by the presence of a low-abundant urinary metabolite.

Finally, hGH reference values for urine were estimated by analyzing 95 doping control routine samples with Kit 1 and 2 of the GH isoform assay. While in most subjects, the measured values were in the low pg/mL range, also concentrations significantly higher than 100 pg/mL were detected in about 18% of the samples. But in contrast to the somatrogon post-administration urine samples, the rhGH concentrations determined with Kit 1 and 2 were found to be comparable, indicating that the Rec2/Rec1 ratio could potentially be employed to flag urine specimens for subsequent LC-HRMS/MS analysis.

## Supplementary Information

Supplementary Information.

## Data Availability

All MS data generated during the current study are available from the corresponding author on reasonable request.
