# Supplementary material for: Detection of the GH analogue somatrogon in doping control urine samples by means of LC-HRMS/MS
Source: Sci Rep. 2025 Apr 16;15:13160. doi: 10.1038/s41598-025-96361-4 (PMC12003886; doi:10.1038/s41598-025-96361-4)
Supplement: Supplementary file 1 — Supplementary Information. [file 41598_2025_96361_MOESM1_ESM.docx]

Supplementary File

**Detection of the GH analogue somatrogon in doping control urine samples by means of LC-HRMS/MS**

K. Walpurgis^1^, A. Thomas^1^, A. Rauer^1^, B. Majer^1^, M. Sato^2^, M. Okano^2^, M. Al-Jaber^3^, W. Abushareeda^3^, C.-G. Arsene^4^, H. Geyer^1, 5^, M. Thevis^1, 5^

^1^Institute of Biochemistry/Center for Preventive Doping Research, German Sport University Cologne, Cologne, Germany

^2^Anti-Doping Laboratory, LSI Medience Corporation, Tokyo, Japan

^3^Anti-Doping Lab Qatar, Doha, Qatar

^4^Physikalisch-Technische Bundesanstalt (PTB), Braunschweig, Germany

^5^European Monitoring Center for Emerging Doping Agents (EuMoCEDA), Cologne/Bonn, Germany

**Overview:**

**Supplementary Methods 1:** Extraction of somatrogon from urine by solid-phase extraction (SPE)

**Supplementary Figure 1:** Extracted ion chromatograms of a urine sample fortified with
10 ng/mL of somatrogon, which was prepared with A) ultrafiltration and B) SPE prior to affinity purification, tryptic digestion, and LC-HRMS/MS.

**Supplementary Figure 2:** Stability of somatrogon (25 ng/mL) in urine samples (1 male (m), 1 female (f)) stored over a period of 14 days at 4 °C and RT.

**Supplementary Figure 3:** Unedited Western blot image of Figure 5.

**Supplementary Table 1:** Tryptic peptides of somatrogon detected by means of LC-HRMS/MS.

**Supplementary Table 2:** Urinary hGH reference values determined with the hGH isoform differential immunoassays.

**Supplementary Methods 1:** Extraction of somatrogon from urine by solid-phase extraction (SPE)

Of each sample, 2 mL of urine were fortified with 200 µL of ACN and centrifuged for 5 min at 4,000 x *g*. For SPE, HLB Oasis cartridges (3 ccm, 60 mg) were employed and equilibrated with 2 mL of ACN and 2 mL of deion. water before applying the supernatants of the samples. After washing the cartridges with 2 mL deion. water, they were eluted with 1.2 mL of a mixture of ACN and water (80/20). Sample extracts were evaporated in a vacuum concentrator, reconstituted with 200 µL of PBS and subjected to affinity purification, tryptic digestion, and LC-HRMS/MS as described in section 2.3 of the manuscript.

**Supplementary Figure 1:** Extracted ion chromatograms of a urine sample fortified with 10 ng/mL of somatrogon, which was prepared with A) ultrafiltration and B) SPE prior to affinity purification, tryptic digestion, and LC-HRMS/MS.

**
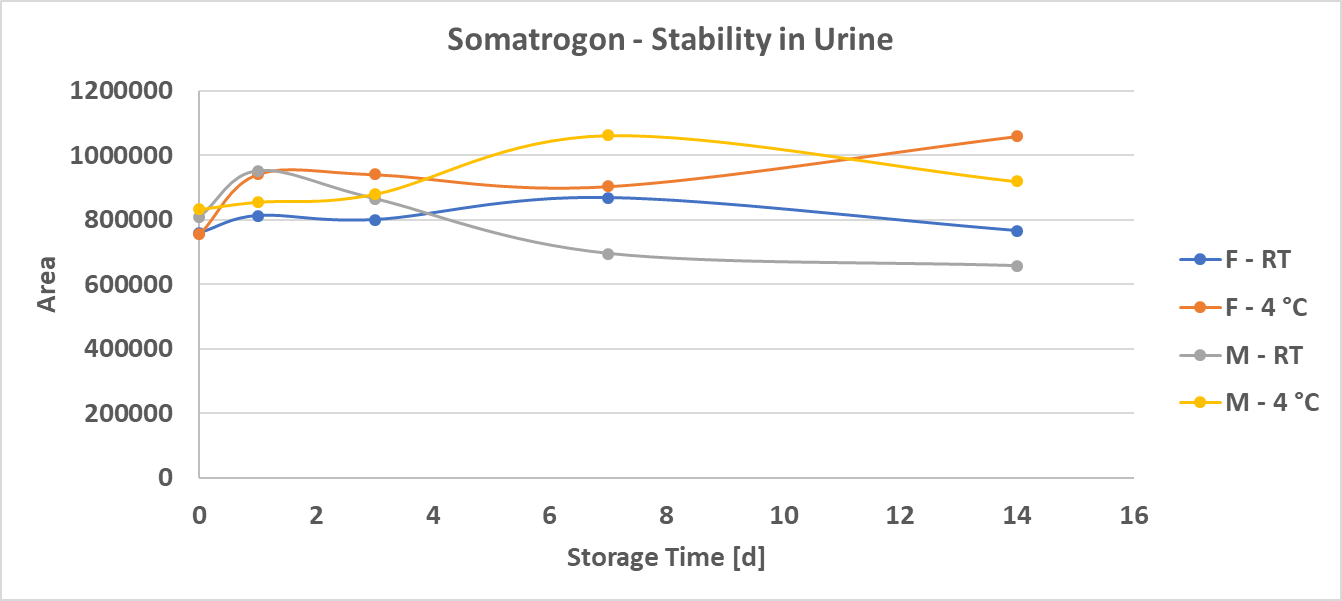
**

**Supplementary Figure 2:** Stability of somatrogon (25 ng/mL) in urine samples (1 male (m), 1 female (f)) stored over a period of 14 days at 4 °C and RT.

**
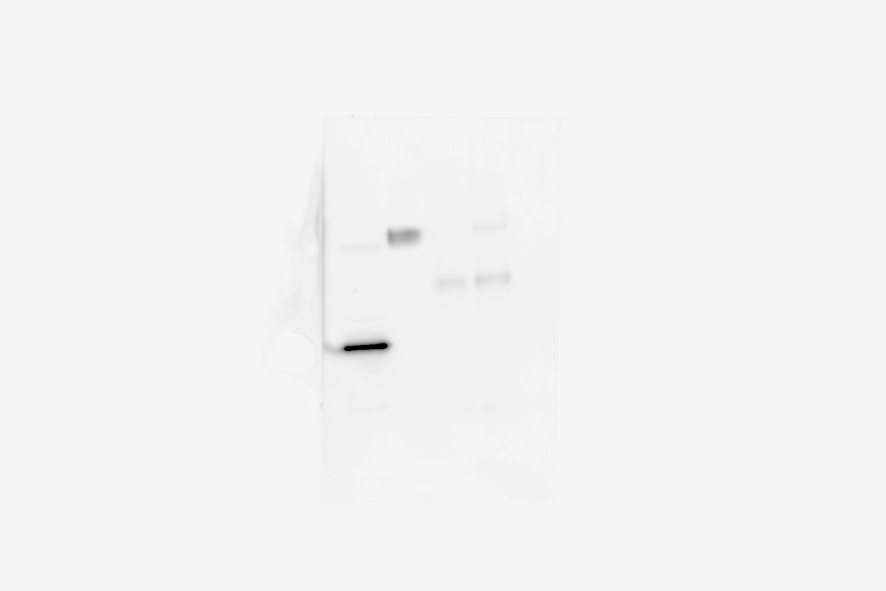
**

**Supplementary Figure 3:** Unedited Western blot image of Figure 5.

**Supplementary Table 1:** Tryptic peptides of somatrogon detected by means of LC-HRMS/MS

**Supplementary Table 2:** Urinary hGH reference values determined with the hGH isoform differential immunoassays.
